# Association of COVID-19 Stay-at-Home Orders With 1-Year Weight Changes

**DOI:** 10.1001/jamanetworkopen.2022.17313

**Published:** 2022-06-16

**Authors:** Rena R. Wing, Kripa Venkatakrishnan, Emily Panza, Oscar C. Marroquin, Kevin E. Kip

**Affiliations:** 1 Weight Control and Diabetes Research Center, The Miriam Hospital, Providence, Rhode Island; 2 Department of Psychiatry and Human Behavior, Alpert Medical School of Brown University, Providence, Rhode Island; 3 Clinical Analytics, University of Pittsburgh Medical Center, Pittsburgh, Pennsylvania

## Abstract

This cohort study assesses mean changes in weight and body mass index in adults during the years before and after stay-at-home orders during the COVID-19 pandemic.

## Introduction

Efforts to slow the transmission of COVID-19 through stay-at-home mandates and shutdown of public places may have led to weight gain and increased rates of obesity. A recent survey^1^ found that 60% of adults reported gaining a mean of 5.6 kg in bodyweight, whereas a meta-analysis^2^ suggested smaller weight gains, at 1.57 kg. Such inconsistent findings may be owing to small samples, self-report, and/or cross-sectional designs. We sought to examine changes in weight and body mass index (BMI; calculated as weight in kilograms divided by height in meters squared) associated with the COVID-19 shutdown using objective weight measures from electronic medical records (EMR) in a large sample of more than 100 000 adults and a within-individual design comparing changes during the year after the COVID-19 shutdown relative to a control period prior to COVID-19.

## Methods

This cohort study was approved by the University of Pittsburgh Medical Center (UPMC) Quality Improvement Review Committee and Institutional Review Board as an exempt protocol, and all data were deidentified. This study followed the Strengthening the Reporting of Observational Studies in Epidemiology (STROBE) reporting guideline for cohort studies.

Using data from the UPMC EMR system, we determined mean changes in weight and BMI in adults who had at least 2 BMI measures at ambulatory visits during both the year after the shutdown (March 16, 2020, to November 12, 2021) and the year immediately prior to the shutdown (January 1, 2018, to March 15, 2020). We also report a sensitivity analysis using only in-person weight measures. We compared the proportion with clinically significant changes in weight,^3^ defined as at least 5% change in weight or comparable 2-unit change in BMI, compared with individuals whose weight was stable, and analyzed differences among important subgroups. More information about participants and methods is provided in the eMethods in the Supplement. Race and ethnicity were based on patient self-report, as documented in the EMR. Race consisted of the categories Alaska Native, American Indian, Asian, Black, Filipino, Indian, Native Hawaiian, Pacific Islander and White. Ethnicity consisted of Hispanic and non-Hispanic. Race and ethnicity were included because they have been associated with obesity and the incidence of COVID-19.

Mean changes in weight and BMI during the preshutdown and the postshutdown periods were computed, and paired *t* tests were used to compare the changes during the 2 time periods. McNemar-Bowker tests were used to compare the percentage of patients with clinically significant changes in weight and BMI during the 2 periods. Given the large sample size and resulting high statistical power, significance was set at 2-sided *P* < .0001. All analyses were performed using SAS statistical software version 9.4 (SAS Institute) and were conducted between November 18 and December 13, 2021.

## Results

We studied 102 889 adults (mean [SD] age, 56.4 [18.2] years; 64% women; 8% Black and 90% non-Hispanic White; mean [SD] BMI, 30.8 [7.3]). The mean (SD) time between time 0 and time 1 (preshutdown) was 10.7 (2.5) months and between time 2 and 3 (postshutdown) was 10.6 (2.5) months.

Participants had statistically significant increases in weight during the preshutdown year (mean change, 0.18 [95% CI, 0.15 to 0.22] kg) and postshutdown year (mean change, 0.22 [95% CI, 0.19 to 0.26] kg), but the difference between the preshutdown and postshutdown changes was not significant (difference, 0.04 [95% CI, −0.01 to 0.10] kg; *P* = .11) (Table). The sensitivity analysis including only patients with all 4 measures assessed in-person found significantly less weight gain in the postshutdown interval vs preshutdown interval (Table). The percentage of individuals who remained weight-stable decreased by 2% from the preshutdown to postshutdown periods, whereas the percentage who either gained or lost 5% increased by approximately 0.7% (Figure). Changes in weight from preshutdown to postshutdown periods did not differ among subgroups. Results for BMI were similar (Table).

**Table.** Weight and BMI Outcomes During Preshutdown and Postshutdown Intervals

Outcome	Patients, No.	Preshutdown interval			Postshutdown interval			Postshutdown change – preshutdown change	
		Mean (SD)		Change, mean (95% CI)	Mean (SD)		Change, mean (95% CI)		
		Time 0	Time 1		Time 2	Time 3		Mean (95% CI)	*P* value^a^
**Primary analysis (N = 102 889)** ^b^									
Body weight, kg	98 308	86.7 (23.0)	86.9 (23.0)	0.18 (0.15 to 0.22)	86.7 (22.9)	87.0 (23.3)	0.22 (0.19 to 0.26)	0.04 (−0.01 to 0.10)	.11
BMI	102 889	30.8 (7.3)	30.9 (7.3)	0.10 (0.08 to 0.11)	30.8 (7.3)	30.9 (7.5)	0.10 (0.09 to 0.11)	0.00 (−0.02 to 0.02)	.84
**Sensitivity analysis (n = 83 678)** ^c^									
Body weight, kg	80 436	86.4 (22.9)	86.7 (22.9)	0.28 (0.25 to 0.32)	86.8 (22.8)	86.9 (23.3)	0.09 (0.05 to 0.13)	−0.20 (−0.25 to −0.14)	<.001
BMI	83 678	30.6 (7.2)	30.8 (7.2)	0.13 (0.12 to 0.15)	30.8 (7.2)	30.8 (7.4)	0.05 (0.04 to 0.06)	−0.08 (−0.11 to −0.06)	<.001

Abbreviation: BMI, body mass index (calculated as weight in kilograms divided by height in meters squared).

^a^ Assessed by paired *t* test.

^b^ Includes patients with at least 2 BMI measures before and after the shutdown.

^c^ Includes patients with at least 2 BMI measures before and after the shutdown at in-person visits (telemedicine visits excluded).

**Figure.**
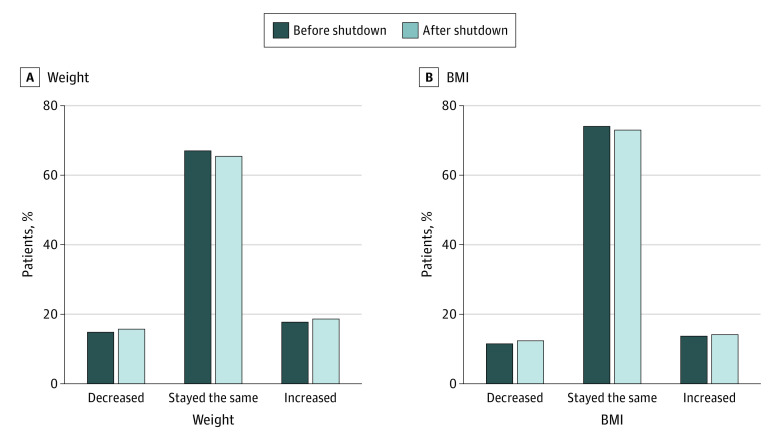
Patients With Clinically Significant Changes in Weight or Body Mass Index (BMI) During the Preshutdown and Postshutdown Intervals Clinical significance was defined as 5% change in weight and 2-unit change in BMI (calculated as weight in kilograms divided by height in meters squared). Assessed using McNemar-Bowker test, weight: *P* < .001; BMI: *P* < .001.

## Discussion

This cohort study found that changes in weight or BMI seen after the COVID-19 shutdown were not significantly greater than those occurring during the preshutdown period in this large sample of adults treated in outpatient settings within a health care system. Differences between our results and prior studies may relate to methodological differences. Many prior studies used self-report and self-selected participants^2^ or reported weight gain over short intervals.^4^ The use of EMR data, a within-individual design, and the comparison of postshutdown changes with those seen during a similar period before the COVID-19 shutdown allowed us to evaluate longer-term changes, interpret changes associated with the shutdown vs changes over time, and to control for key fixed (non–time-varying) covariates (eg, sex, race).

Limitations of our study include the lack of standardization of measures in the EMR,^5^ inability to examine causes of weight change (eg, physical activity, dietary intake), and lack of racial and ethnic diversity in this sample. However, weight changes did not differ between racial groups, consistent with prior research.^6^ Our results may not generalize to all adults, since only individuals who seek medical care are captured via EMR data. These findings should help to mitigate public health concerns that COVID-19 shutdown orders led to weight gain in adults.

## Notes

Supplement.
eMethods.

## References

[1] Glazer SA, Vallis M. Weight gain, weight management and medical care for individuals living with overweight and obesity during the COVID-19 pandemic (EPOCH study). Obes Sci Pract. Published online January 18, 2022. doi:10.1002/osp4.591 PMC9535671 36238224

[2] Bakaloudi DR, Barazzoni R, Bischoff SC, Breda J, Wickramasinghe K, Chourdakis M. Impact of the first COVID-19 lockdown on body weight: a combined systematic review and a meta-analysis. Clin Nutr. 2021;S0261-5614(21)00207-7. doi:10.1016/j.clnu.2021.04.015 34049749 PMC8056819

[3] Jensen MD, Ryan DH, Donato KA, ; American College of Cardiology/American Heart Association Task Force on Practice Guidelines, Obesity Expert Panel, 2013. Executive summary: guidelines (2013) for the management of overweight and obesity in adults: a report of the American College of Cardiology/American Heart Association Task Force on Practice Guidelines and the Obesity Society published by the Obesity Society and American College of Cardiology/American Heart Association Task Force on Practice Guidelines—based on a systematic review from the The Obesity Expert Panel, 2013. Obesity (Silver Spring). 2014;22(suppl 2):S5-S39. doi:10.1002/oby.20821 24961825

[4] Lin AL, Vittinghoff E, Olgin JE, Pletcher MJ, Marcus GM. Body weight changes during pandemic-related shelter-in-place in a longitudinal cohort study. JAMA Netw Open. 2021;4(3):e212536-e212536. doi:10.1001/jamanetworkopen.2021.2536 33749764 PMC7985720

[5] Gianfrancesco MA, Goldstein ND. A narrative review on the validity of electronic health record-based research in epidemiology. BMC Med Res Methodol. 2021;21(1):234. doi:10.1186/s12874-021-01416-5 34706667 PMC8549408

[6] Mulugeta W, Desalegn H, Solomon S. Impact of the COVID-19 pandemic lockdown on weight status and factors associated with weight gain among adults in Massachusetts. Clin Obes. 2021;11(4):e12453. doi:10.1111/cob.12453 33855789 PMC8250379

