# Supplementary material for: Association of COVID-19 Stay-at-Home Orders With 1-Year Weight Changes
Source: JAMA Netw Open. 2022 Jun 16;5(6):e2217313. doi: 10.1001/jamanetworkopen.2022.17313 (PMC9204539; doi:10.1001/jamanetworkopen.2022.17313)
Supplement: Supplement. — eMethods. [file jamanetwopen-e2217313-s001.pdf]

# Supplemental Online Content

Wing RR, Venkatakrishnan K, Panza E, Marroquin OC, Kip KE. Association of COVID-19 stay-at-home orders with 1-year weight changes. *JAMA Netw Open*. 2022;5(6):e2217313. doi:10.1001/jamanetworkopen.2022.17313

**eMethods.**

This supplemental material has been provided by the authors to give readers additional information about their work.

© 2022 Wing RR et al. *JAMA Network Open.*

## eMethods.

**Data Collection.** Data were extracted from the UPMC EMR system and included 22 hospitals whose data have been harmonized in the UPMC Clinical Data Warehouse. The UPMC is an academic health care system primarily in the central and western parts of Pennsylvania. The data include basic demographic data (age, gender, self-reported race/ethnicity, marital status) and clinical measures from each patient's visits. Each patient also has a score on the Area Deprivation Index (ADI), based on census data related to education, income/employment, housing, and household characteristics. The highest 15% for ADI represent individuals from the most deprived areas[1]. Our study received formal ethics approval by the UPMC Ethics and Quality Improvement Review Committee (Project ID 2882).

**Methods**. Since the shutdown in the Pittsburgh area occurred on or around March 16, 2020, PRE- and POST-Shutdown were defined relative to this date. Using ambulatory visits only (excluding in-hospital or ER visits), EMR records were used to identify all adults (age 18 or over) who met the following eligibility criteria: PRE-Shutdown: had 2 or more BMI measures prior to 3/16/2020, with greater than 90 days between the two measures and the most recent measure within 90 days of 3/16/2020; POST-Shutdown: had 2 or more BMI measures on or after 3/16/2020 with greater than 90 days between the measures and the first measure occurring between 3/16/2020 and 5/31/2020. For patients with more than two qualifying measures PRE- and/or POST-Shutdown, the records that were closest to one year apart were selected. Since the shut-down led to restrictions on in-person visits and increased use of telemedicine for these visits, we also conducted a sensitivity analysis using only patients where all 4 measures were done in person.

**Statistical Analysis**. Analyses were based on all usable data, excluding only extreme values (top and bottom .05%) considered biologically implausible. Mean changes (differences) in weight and BMI measures between the two PRE measures (Time 0 and 1) and two POST measures (Time 2 and 3) were compared by use of paired t-tests. Subsequent categorical analyses used the McNemar-Bowker test to compare the proportion of individuals with clinically defined increases, no change, or decreases in these

© 2022 Wing RR et al. *JAMA Network Open.*

parameters during the PRE and POST periods. Based on prior studies showing that a 5% change in body weight produced clinically significant changes in health parameters[2], we used this criterion for weight and a comparable criterion of a 2-unit change in BMI. Subgroup analyses were performed to compare changes in weight and BMI by age group (18-35; 36-50; 51-60; 61-70 and >71 years), baseline BMI (<18.5, 18.5-<25; 25-<30, 30-<35 and 35 or higher), race (white; black; other) ADI, and gender.

**Participants**. All patients who were in the UPMC EMR system and had 2 or more ambulatory visits (excluding emergency room and inpatient visits) during the period PRE-shutdown and the period POST-shutdown were considered for this analysis. Participants in this analysis had been seen mainly in internal medicine or family medicine settings. Approximately 50% of these patients were on hypertensive medications and 20% of these patients had type 2 diabetes. We did not exclude patients who had diseases that could lead to weight losses or gains, due to the difficulty of selecting such diseases, the time course of weight changes that may occur, and the effect of medications to treat these conditions on changes in weight.

## References


1. Kind AJ, Jencks S, Brock J, et al. Neighborhood socioeconomic disadvantage and 30-day rehospitalization: a retrospective cohort study. *Ann Intern Med.* 2014;161(11):765-774.
2. Jensen MD, Ryan DH, Donato KA, et al. Executive summary: guidelines (2013) for the management of overweight and obesity in adults. *Obesity.* 2014;22(S2):S5-S39.


© 2022 Wing RR et al. *JAMA Network Open.*
